# Supplementary material for: Reproducible and transparent research practices in published neurology research
Source: Res Integr Peer Rev. 2020 Feb 28;5:5. doi: 10.1186/s41073-020-0091-5 (PMC7049215; doi:10.1186/s41073-020-0091-5)
Supplement: Supplementary file 1 — Additional file 1: Table S1. Additional Characteristics of Reproducibility in Neurology Studies. [file 41073_2020_91_MOESM1_ESM.docx]

| **Supplemental 1: Additional Characteristics of Reproducibility in Neurology Studies** | | | |
| --- | --- | --- | --- |
| **Characteristics** | | **Variables** | |
|  | | **N (%)** | **95% CI** |
| **Conflict of Interest Statement**  **(N=389)** | Statement, one or more conflicts of interest | 47 (12.1) | 8.9-15.3 |
|  | Statement. no conflict of interest | 216 (55.5) | 50.7-60.4 |
|  | No conflict of interest statement | 126 (32.4) | 27.8-37.0 |
|  | | | |
| **Data Availability (N=271)** | Statement, some data are available | 25 (9.2) | 6.0-12.5 |
|  | Statement, data are not available | 1 (0.4) | 0-1.1 |
|  | No data availability statement | 245 (90.4) | 87.1-93.7 |
|  | | | |
| **Material Availability (N=255)** | Statement, some materials are available | 24 (9.4) | 6.6-12.3 |
|  | Statement, materials are not available | 0 | 0 |
|  | No materials availability statement | 231 (90.6) | 87.7-93.4 |
|  | | | |
| **Protocol Availability (N=271)** | Full Protocol | 2 (0.7) | 0-1.6 |
|  | No Protocol | 269 (99.3) | 98.4-100 |
|  | | | |
| **Analysis Scripts (N=271)** | Statement, some analysis scripts are available | 2 (0.7) | 0-1.6 |
|  | Statement, analysis scripts are not available | 0 | 0 |
|  | No analysis script availability statement | 269 (99.3) | 98.4-100 |
|  | | | |
| **Replication Studies**  **(N=271)** | Reports Replication Study | 0 | 0 |
|  | No Clear Statement | 271 (100) | 0 |
|  |  |  |  |
| **Open Access**  **(N=400)** | Yes ­ found via Open Access Button | 168 (57.1) | 52.3-62.0 |
|  | Yes ­ found article via other means | 5 (1.7) | 0.4-3.0 |
|  | Could not access through paywall | 227 (77.2) | 73.1-81.3 |
|  |  |  |  |
| **Cited in a Systematic Review/**  **Meta-Analysis (a) (N=275)** | No Citations | 223 (81.1) | 77.3-84.9 |
|  | A Single Citation | 36 (13.1) | 9.8-16.4 |
|  | One to Five Citations | 15 (5.5) | 3.2-7.7 |
|  | More Than 5 Citations | 1 (0.4) | 0-1.0 |
| Abbreviations: CI, Confidence Interval. a - No studies were explicitly excluded from the systematic reviews or meta-analyses that cited the original article. | | | |
|  |  |  |  |
