# Supplementary material for: Reproducible and transparent research practices in published neurology research
Source: Res Integr Peer Rev. 2020 Feb 28;5:5. doi: 10.1186/s41073-020-0091-5 (PMC7049215; doi:10.1186/s41073-020-0091-5)
Supplement: Supplementary file 2 — Additional file 2: Table S2. Additional Characteristics of Reproducibility in Neurology Studies. [file 41073_2020_91_MOESM2_ESM.docx]

| **Supplemental 2: Additional Characteristics of Reproducibility in Neurology Studies** | | | |
| --- | --- | --- | --- |
| **Characteristics** | | **Variables** | |
|  | |  |  |
| **Pre-Registration (N=271)** | Statement, says was pre-­registered | 10 (3.7) | 1.8-5.5 |
|  | Statement, says was not pre-registered | 0 | 0 |
|  | No, there is no pre-registration statement | 261 (96.3) | 94.5-98.2 |
|  |  |  |  |
| **Test Subjects**  **(N=389)** | Animals | 68 (17.5) | 13.8-21.2 |
|  | Humans | 199 (51.2) | 46.3-56.1 |
|  | Both | 0 | 0 |
|  | Neither | 122 (31.4) | 26.8-35.9 |
|  |  |  |  |
| **Country of Journal Publication**  **(N=389)** | United States | 220 (56.6) | 51.7-61.4 |
|  | UK | 96 (24.7) | 20.5-28.9 |
|  | Netherlands | 26 (6.7) | 4.2-9.1 |
|  | Germany | 10 (2.6) | 1.0-4.1 |
|  | Ireland | 12 (3.1) | 1.4-4.8 |
|  | Switzerland | 4 (1.0) | 0-2.0 |
|  | Taiwan | 1 (0.3) | 0-0.8 |
|  | Other (a) | 20 (5.1) | 3.0-7.3 |
|  |  |  |  |
| **Country of Corresponding Author**  **(N=290)** | United States | 127 (32.6) | 28.1-37.2 |
|  | China | 26 (6.7) | 4.2-9.1 |
|  | UK | 28 (7.2) | 4.7-9.7 |
|  | Netherlands | 9 (2.3) | 0.8-3.8 |
|  | Turkey | 8 (2.1) | 0.7-3.4 |
|  | France | 11 (2.8) | 1.2-4.5 |
|  | Canada | 16 (4.1) | 2.2-6.1 |
|  | Italy | 20 (5.1) | 3.0-7.3 |
|  | Brazil | 11 (2.8) | 1.2-4.5 |
|  | Australia | 19 (4.9) | 2.8-7.0 |
|  | Other | 114 (29.3) | 24.8-33.8 |
