# Supplementary material for: Reproducible and transparent research practices in published neurology research
Source: Res Integr Peer Rev. 2020 Feb 28;5:5. doi: 10.1186/s41073-020-0091-5 (PMC7049215; doi:10.1186/s41073-020-0091-5)
Supplement: Supplementary file 3 — Additional file 3: Table S3. Additional Characteristics of Reproducibility in Neurology Studies. [file 41073_2020_91_MOESM3_ESM.docx]

| **Supplemental 3: Additional Characteristics of Reproducibility in Neurology Studies** | | |
| --- | --- | --- |
| **Characteristics** | | **Variables** |
|  | | **N** |
| **Material Availability** | Personal or institutional | 2 |
|  | Supplementary information hosted by the journal | 16 |
|  | Online third party | 5 |
|  | Upon Request | 1 |
|  | Yes, material was accessible | 17 |
|  | No, material was not accessible | 7 |
|  |  |  |
| **Data Availability** | Personal or institutional | 1 |
|  | Supplementary journal information | 12 |
|  | Online third party | 6 |
|  | Upon Request | 5 |
|  | Other (b) | 1 |
|  | Yes, data could be accessed and downloaded | 6 |
|  | No, data count not be accessed and downloaded | 19 |
|  | Yes, data files were clearly documented | 3 |
|  | No, data files were not clearly documented | 3 |
|  | Yes, data files contain all raw data | 2 |
|  | No, data files do not contain all raw data | 3 |
|  | Unclear if all raw data was available | 1 |
|  |  |  |
| **Pre-Registration** | Yes, there was a pre-registration | 10 |
|  | Registered on ClinicalTrials.Gov | 6 |
|  | Registered on Other | 4 |
|  | Hypothesis was pre-registered | 2 |
|  | Methods were pre-registered | 10 |
|  | Analysis plan was pre-registered | 8 |
